# Supplementary material for: Iterative improvement in the automatic modular design of robot swarms
Source: PeerJ Comput Sci. 2020 Dec 7;6:e322. doi: 10.7717/peerj-cs.322 (PMC7924708; doi:10.7717/peerj-cs.322)
Supplement: Supplemental Information 3 [file peerj-cs-06-322-s003.zip › argos3/doc/api/standalone/a00612_source.html]

ARGoS: plugins/simulator/physics\_engines/dynamics2d/dynamics2d\_multi\_body\_object\_model.h Source File


- Main Page
- Related Pages
- Namespaces
- Classes
- Files

- File List
- File Members

# plugins/simulator/physics\_engines/dynamics2d/dynamics2d\_multi\_body\_object\_model.h

Go to the documentation of this file.

```
00001 
00007 #ifndef DYNAMICS2D_MULTI_BODY_OBJECT_MODEL_H
00008 #define DYNAMICS2D_MULTI_BODY_OBJECT_MODEL_H
00009 
00010 namespace argos {
00011    class CDynamics2DMultiBodyObjectModel;
00012 }
00013 
00014 #include <argos3/plugins/simulator/physics_engines/dynamics2d/dynamics2d_model.h>
00015 #include <argos3/plugins/simulator/physics_engines/dynamics2d/chipmunk-physics/include/chipmunk.h>
00016 
00017 namespace argos {
00018 
00044    class CDynamics2DMultiBodyObjectModel : public CDynamics2DModel {
00045 
00046    public:
00047 
00048       struct SBody {
00049          cpBody* Body;
00050          cpVect  OffsetPos;
00051          cpFloat OffsetOrient;
00052          Real    Height;
00053          SBody(cpBody* pt_body,
00054                const cpVect& t_offset_pos,
00055                cpFloat t_offset_orient,
00056                Real f_height);
00057       };
00058 
00059    public:
00060 
00067       CDynamics2DMultiBodyObjectModel(CDynamics2DEngine& c_engine,
00068                                       CComposableEntity& c_entity);
00069 
00074       virtual ~CDynamics2DMultiBodyObjectModel();
00075 
00080       inline CComposableEntity& GetComposableEntity() {
00081          return m_cEntity;
00082       }
00083 
00088       inline const CComposableEntity& GetComposableEntity() const {
00089          return m_cEntity;
00090       }
00091 
00096       inline SBody& GetBody(size_t i) {
00097          return m_vecBodies[i];
00098       }
00099       
00104       inline const SBody& GetBody(size_t i) const {
00105          return m_vecBodies[i];
00106       }
00107       
00108       virtual void Reset();
00109 
00110       virtual void MoveTo(const CVector3& c_position,
00111                           const CQuaternion& c_orientation);
00112 
00113       virtual void CalculateBoundingBox();
00114 
00115       virtual void UpdateFromEntityStatus()  = 0;
00116 
00117       virtual bool IsCollidingWithSomething() const;
00118 
00143       virtual void AddBody(cpBody* pt_body,
00144                            const cpVect& t_offset_pos,
00145                            cpFloat t_offset_orient,
00146                            Real f_height);
00147 
00148    private:
00149 
00150       CComposableEntity& m_cEntity;
00151       std::vector<SBody> m_vecBodies;
00152    };
00153 
00154 }
00155 
00156 #endif
```

---

Generated on 10 Jul 2018 for ARGoS by 
 1.6.1
